# Bio-effects of engineering nanomaterials NiFe-based LDHs on ryegrass-soil system

**DOI:** 10.1007/s44307-026-00114-x

**Published:** 2026-06-24

**Authors:** Haocheng Xu, Xiaoqian Jiang, Chuntao He, Yutao Peng, Guorong Xin, Xiaoyun Li

**Affiliations:** 1 State Key Laboratory of Biocontrol, Guangdong Provincial Key Laboratory of Plant Stress Biology, School of Agriculture and Biotechnology, Sun Yat-Sen University, Shenzhen, Guangdong 518107 PR China; 2 School of Agriculture and Biotechnology, Sun Yat-Sen University, Shenzhen, Guangdong 518107 PR China

**Keywords:** Engineering nanomaterials, Plant-soil system, Plant growth, Enzymatic activity, Microbial community structure

## Abstract

NiFe-based layered double hydroxides (LDHs), as engineered nanomaterials (ENMs), are widely utilized in industrial applications, environmental remediation, and soil improvement. However, the biological impacts of such ENMs on plant-soil systems remain insufficiently explored. A 50-day soil cultivation experiment was conducted to assess the effects of two-dimensional and three-dimensional NiFe-based LDHs (2D NiFe-LDHs and 3D NiFeS-LDHs) on ryegrass growth and soil ecosystem. Generally, morphology and structure differences influence the biological effects of NiFe-based LDHs. 2D NiFe-LDHs promote ryegrass growth at specific concentrations, whereas 3D NiFeS-LDHs exhibit significant inhibitory effects on ryegrass growth. NiFe-based LDHs directly impacted soil geochemistry. 3D NiFeS-LDHs decreased soil pH while increasing electrical conductivity and soil organic carbon. 2D NiFe-LDHs inhibited soil sucrase (S-SC) activity, but enhanced soil urease (S-UE) activity. Conversely, NiFeS-LDHs inhibited catalase (S-CAT) activity and S-UE activity, while promoting neutral phosphatase (S-NP) activity However, as concentrations increased, the effects of 2D NiFe-LDHs and 3D NiFeS-LDHs on soil properties and enzyme activities became more similar. Both NiFe-based LDHs altered the diversity of soil bacterial and fungal communities, with 3D NiFeS-LDHs exerting a stronger inhibitory effect on fungal diversity at higher concentrations. The structural differences in NiFe-based LDHs exert a distinct and substantial impact on the composition of soil microbial communities and soil enzyme activities. The findings provide critical insights into bio-effects of NiFe-based LDHs, which provide a theoretical basis for the study of potential ecological risks in soil.

**Supplementary Information:**

The online version contains supplementary material available at 10.1007/s44307-026-00114-x.

## Introduction

Nanomaterials demonstrate significant application potential due to their unique properties, with applications in biomedicine, photoelectrocatalysis, agricultural chemicals, and environmental remediation (Selmani et al. 2022; Xu et al. 2023). The unique properties of engineering nanomaterials (ENMs) drive their widespread use, resulting in a steady increase in production volume. The rise in production volume has led to an increase in ENMs emissions into the environment (Benn and Westerhoff 2008). Layered double hydroxides (LDHs) are two-dimensional (2D) inorganic metal nanomaterials (ENMs) with the chemical formula [M^2+^_1-x_ M^3+^_x_(OH)_2_] A^n^_x/n_·yH_2_O, where M^2+^ and M^3+^ represent 2 + and 3 + metal cations, respectively, and A^n–^ denotes an interlayer anion (Zhang and Liu 2022). LDHs have the characteristics of high specific surface area, anion exchange capacity, and thermal stability, making it an ideal choice for various environmental and industrial applications. According to the Chinese chemical industry standard (HG/T 5549–2019), LDHs is recognized as an environmentally friendly adsorbent material (Zhang et al. 2022). The hierarchical porous composite formed by NiFe LDHs and MoS^2−^ ions exhibited ultra-high adsorption capacities of 462, 299, and 128 mg·g^−1^ for Hg^2+^, Pb^2+^, and Cu^2+^, respectively (Zhang et al. 2022). NiFe LDHs was able to remove 91.1% of Congo Red (CR) and 90.7% of Rhodamine 6G under visible light irradiation (Suppaso et al. 2021). Zubair et al. developed a novel S/NiFe LDHs composite, consisting of starch and NiFe LDHs in a 1:1 ratio, which demonstrated a high adsorption capacity for methyl orange dye removal from water (Zubair et al. 2018). LDHs proved effective not only for water remediation but also for soil restoration. The application of CaAl-LDHs in farmland effectively reduced soil available Cu, Zn, Cd, and Pb, with fixation efficiencies of 30.15%, 67.30%, 57.80%, and 38.71%, respectively. It also lowered heavy metal content in plant roots without impacting the growth or yield of buckwheat (Sun et al. 2023). LDHs not only effectively removed heavy metals from soil but also functioned as a soil amendment, serving in the preparation of slow-release multifunctional composite nitrogen fertilizers to enhance nitrate assimilation and bind with Zn upon degradation (Gogoi et al. 2022). NiFe-LDHs was also applied as a slow-release carbon source for biological denitrification (Jiang et al. 2018).

In recent years, 3D LDHs have attracted considerable research interest for water treatment due to their larger surface area and more developed porous structure (Li et al. 2016). A study by Zhou et al. found that the sorption capacity of 3D MgAl LDHs for sulfonated lignite in petroleum wastewater was significantly higher (1014.20 mg·g^−1^) compared to 2D MgAl-LDHs (86 mg·g^−1^) (Zhou et al. 2021). The 3D composite material combining GO and NiFe LDHs demonstrated excellent adsorption capacities for CR, MO, and Cr (VI), highlighting its potential to alleviate anionic pollutants in aquatic environments (Zheng et al. 2019). Structural regulation of LDHs significantly enhanced their potential and effectiveness in treating various pollutants, making them more adaptable to diverse environmental applications. LDHs, as an ideal adsorbent for organic pollutants, may cause significant toxicity to organisms when applied on a large scale. Existing research on LDHs toxicity primarily examines its effects on aquatic plants, with limited studies addressing its broader ecological impact. The ecological and environmental hazards of ENM are closely associated with their types and structures. Recently, several researches focused on the toxicity of 2D LDHs to aquatic algae (Qiu et al. 2022; Torbati et al. 2024). However, NiFe-based LDHs ENMs enter various environmental compartments through direct use, accidental release, or indirect waste disposal pathways (Gottschalk et al. 2009). Soil is the primary environmental compartment where NiFe-based LDHs ENMs tend to accumulate. The impact of LDHs on the structure (e.g., richness and evenness) and enzyme activity of terrestrial plants and soil microorganisms after agricultural application remains unknown. The potential risks of NiFe-based LDHs as engineered nanomaterials in natural soil environments stem from their direct interaction with cell surfaces, the dissolution of toxic elements, and the generation of reactive oxygen species (ROS) (Harmsen 2008; Huang et al. 2022).

Italian ryegrass (*Lolium multiflorum* Lam) is a widely cultivated, fast-growing annual grass of the Poaceae family, known for its high biomass, strong regenerative capacity, and resistance to pests and diseases. It serves as a cultivated grass and cover crop with significant economic and environmental value (Zhou et al. 2020). Additionally, ryegrass demonstrates tolerance to and accumulation of heavy metals and nanomaterials (Zhou et al. 2020; Han et al. 2021). These characteristics make ryegrass a suitable model for studying interactions between plants and ENMs. Zhou et al. (Zhou et al. 2020) investigated the impact of TiO_2_ and ZnO nanoparticles on bacteria in ryegrass-cultivated agricultural soil. Vera-Villalobos (Vera-Villalobos et al. 2020) studied the effect of sulfate supply on short-term Al^3+^ toxicity in ryegrass roots.

The ecological and environmental risks associated with LDHs are closely linked to their type and structural characteristics. While existing research predominantly emphasizes the toxicity of LDHs to aquatic organisms, but there is still lack of researches on the effects of LDHs in plant-soil systems. A 50-day soil cultivation experiment was conducted to assess the effects of 2D and 3D NiFe-based LDHs on ryegrass growth and soil ecosystem. This study investigates the biological effects of 2D NiFe-LDHs and 3D NiFeS-LDHs on plant-soil systems, focusing on their impacts on ryegrass as a model plant. Additionally, this study examines the impacts of LDHs on soil microbes and enzymes, as well as the differences between 2D NiFe-LDHs and 3D NiFeS-LDHs. Our findings provide valuable toxicological data for NiFe-based LDHs ENM and support their future applications.

## Materials and methods

### Preparation of NiFe-based LDHs materials

#### Preparation of 2D NiFe-LDHs

2D NiFe-LDHs were prepared via a hydrothermal method. Specifically, 0.01 M FeCl_3_·6H_2_O and 0.03 M Ni(NO_3_)_2_·6H_2_O were dissolved in 60 mL of ultrapure H_2_O. Subsequently, 40 mL of a 2.5 M urea solution was mixed with the metal salt solution and agitated at room temperature for 1 h. The obtained solution was then heated at 120 °C for 24 h. After natural cooling to room temperature, the mixture was centrifuged, thoroughly rinsed with ultrapure water until the filtrate reached pH 7, and the solid NiFe-LDH product was collected. The X-ray powder diffraction (XRD) and scanning Electron Microscope (SEM) images of NiFe-LDHs confirmed the successful preparation of 2D NiFe-LDHs (Fig. S1A and Fig. S2A).

#### Preparation of 3D NiFeS-LDHs

2D NiFe-LDHs (0.5 g) were added to 60 mL of anhydrous ethanol, sonicated for 30 min, and stirred with 0.5 g of thioacetamide. After stirring for 1 h, the mixture was transferred to a hydrothermal reactor and heated at 120 °C for 6 h. The centrifuged product was washed with anhydrous ethanol and ultrapure H_2_O, and then dried in a vacuum oven. The XRD and SEM spectra of NiFeS-LDHs confirmed the successful synthesis of 3D NiFeS-LDHs (Fig. S1B and Fig. S2B). Characterization of the physicochemical properties (specific surface area, pore size, Zeta potential, conductivity) of 2D NiFe-LDHs and 3D NiFeS-LDHs was conducted (Table S1).

#### Structural characterizations

The phase structure of the prepared samples was analyzed by XRD with Cu/Kα_1_ radiation. The microstructure was examined using a Gemini 500 high-resolution thermal field emission scanning electron microscope. Elemental analysis was performed using inductively coupled plasma mass spectrometry (ICP-MS) on an Agilent 7900 ICP-MS instrument.

### Plant culture

#### Soil sample site information

This study used Italian ryegrass (*Lolium multiflorum Lam.*) as a representative plant. The soil was obtained from the surface (0–20 cm) of yellow soil in farmland near the campus (113° 54′ E, 22° 40′ N). After air-drying, the soil was sieved through a 2 mm steel sieve, mixed thoroughly, and stored in dark conditions until use.

#### Plant cultivation

In this experiment, 2 kg of soil was used per pot, with a control group (CK) and treatment groups of 2D NiFe-LDHs/3D NiFeS-LDHs at concentration gradients of 200, 350, 500, 650, and 800 mg/kg. Detailed procedures are available in Supplementary Information (SI), Text S1.

#### Plant harvesting and soil sample collection

After 50 days of cultivation, ryegrass and soil samples were collected to examine the effects of 2D NiFe-LDHs and 3D NiFeS-LDHs on ryegrass growth and the soil ecosystem (soil physicochemical properties, enzyme activity, and microbial community structure). Specific steps are outlined in Supplementary Information (SI), Text S2.

### Growth physiological indicators of ryegrass

After 50 days of exposure to 2D NiFe-LDHs and 3D NiFeS-LDHs nanoparticles, growth physiological indicators such as chlorophyll content, H_2_O_2_ content, superoxide dismutase (SOD), catalase (CAT), and peroxidase (POD) were measured to assess their effects on ryegrass. Details are given in Text S3 in the SI.

#### Determination of Ni and Fe content

Dried leaves or roots were subjected to microwave digestion with 8 mL of nitric acid. The digested samples were then analyzed by ICP-MS (Agilent 7900 ICP-MS).

### Soil physicochemical properties, enzyme activity, and microbial community

#### Physicochemical properties

Soil pH measured in a soil–water suspension using smartCHEM-LAB (SCHL065) at a ratio of 1:2.5 (m:V). Electrical conductivity (EC) was measured in the extraction solution using the smart CHEM-LAB (SCHL065). Total nitrogen (TN) and total carbon (TC) in soil were determined using an elemental analyzer (Thermo Fisher, Thermo Scientific FlashSmart). Available phosphorus (AP) was determined by the molybdenum antimony spectrophotometry method after sodium bicarbonate extraction. Soil organic carbon (SOC) was measured using the potassium dichromate method. Subjected air-dried soil to microwave digestion with 8 mL of reverse aqua regia (HNO_3_: HCl 3:1). The concentrations of Ni, Fe, and total phosphorus (TP) were measured using ICP-MS.

#### Soil enzyme activities

After 50 days of exposure to 2D NiFe-LDHs and 3D NiFeS-LDHs nanoparticles, soil enzyme activities, including sucrase (S-SC), catalase (S-CAT), phosphatase (S-NP), and urease (S-UE), were measured following the instructions of the respective commercial reagent kit (Suzhou Keming Biotechnology Co., Ltd., Suzhou, China) to assess the impact on soil material transformation. Detailed analysis methods are provided in SI, Text S4.

#### Soil microbial community

Soil bacterial community structure was determined by 16S rDNA sequencing. Selected the E.Z.N.A.® Soil DNA Kit to extract total DNA from the soil bacterial microbiome. DNA extraction quality was checked by agarose gel electrophoresis, and DNA concentration was measured using an ultraviolet spectrophotometer. The fungal community structure was analyzed using ITS (Internal Transcribed Spacer) sequencing. Total microbial DNA from soil fungal communities was extracted using the Tiangen Soil and Fecal Genomic DNA Extraction Kit (DP712-02, Tiangen Biotech, Beijing, China) following the bead-beating method, and DNA concentration was quantified using a Qubit fluorometer (Invitrogen, USA) (Table S2). Detailed analysis methods are provided in SI, Text S5.

### Statistical analysis

Statistical analyses were performed using SPSS 27.0 (IBM Corp., USA) and OriginPro version 8.0 (OriginLab Corp., USA). Significant differences among treatments were determined through one-way ANOVA followed by Duncan’s multiple range test at a significance level of *P* < 0.05. Data are expressed as mean ± standard deviation (SD) from quadruplicate measurements.

## Result

### The effect of LDHs on the growth of ryegrass

#### The effect of LDHs on physiological and biochemical indicators of ryegrass

**Fig. 1:**
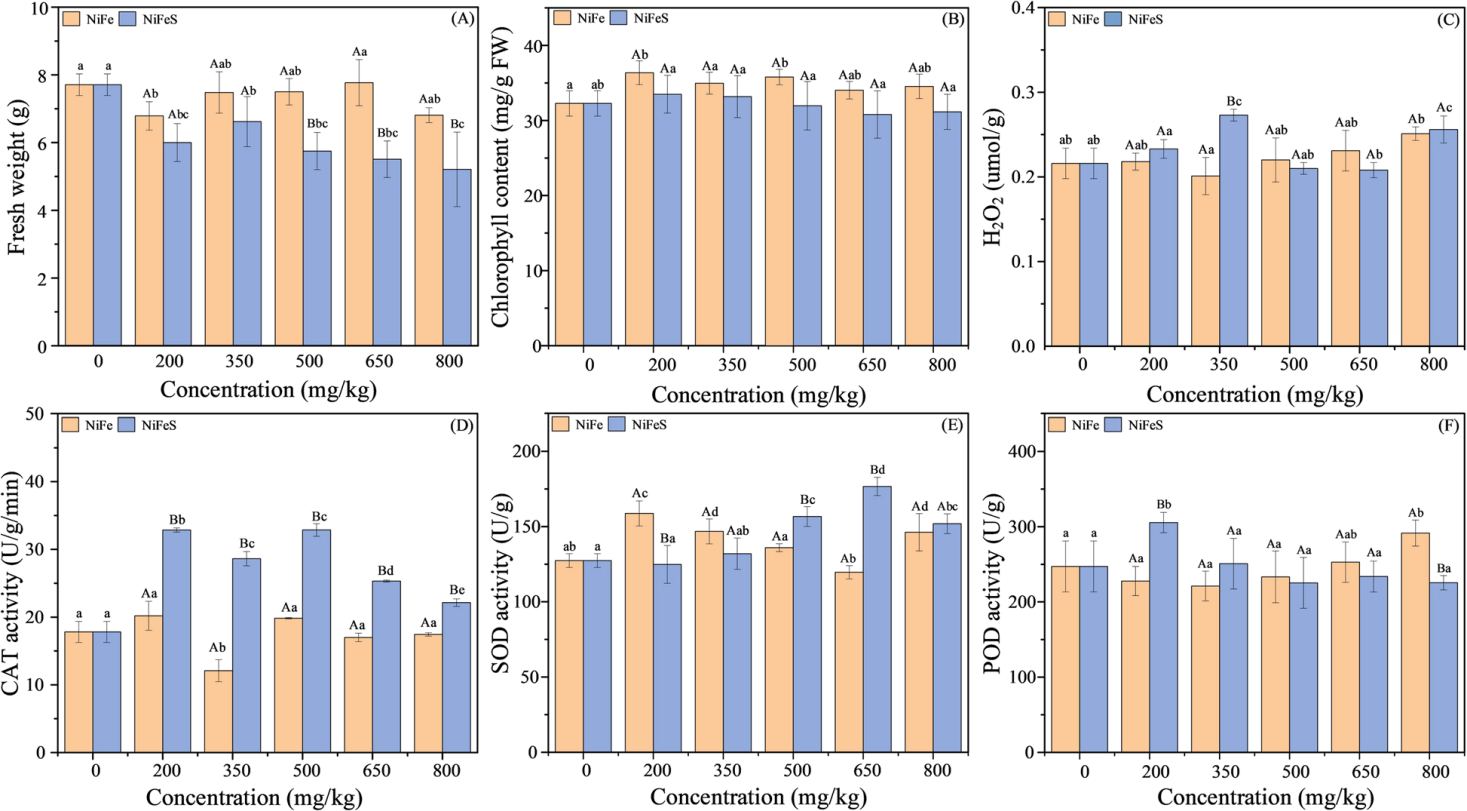
Illustrates the effects of LDH on the fresh weight (**A**), chlorophyll content (**B**), H_2_O_2_ content (**C**), and activities of CAT, SOD, and POD (D-E) in ryegrass. Lowercase letters on the bar chart denote significant differences between treatments, while uppercase letters indicate significant differences between 2D NiFe-LDHs a nd 3D NiFeS-LDHs (*P* < 0.05)

In this experiment, we evaluated the effects of incorporating 2D NiFe-LDHs and 3D NiFeS-LDHs into soil on ryegrass physiological and biochemical parameters, such as chlorophyll content, soluble protein, and hydrogen peroxide levels. As shown in Fig. 1A, the fresh weight of ryegrass was significantly influenced by the type and concentration of NiFe-based LDHs nanomaterials. Both NiFe-LDHs and NiFeS-LDHs exhibited a similar trend, where the fresh weight displayed a three-phase variation characteristic of initial decrease, followed by an increase, and then a subsequent decrease with increasing concentration. Compared with the control group, the fresh weight was significantly inhibited (*P* < 0.05) by 2D NiFe-LDHs at a concentration of 200 mg/kg. In contrast, 3D NiFeS-LDHs caused a significant reduction in fresh weight (*P* < 0.05) at all exposure concentrations.

Figure 1B illustrates the impact of 2D NiFe-LDHs and 3D NiFeS-LDHs on ryegrass chlorophyll content. When 2D NiFe-LDHs concentrations ranged from 200 to 500 mg/kg, the chlorophyll content significantly increased compared to the blank control (CK). However, with increasing 3D NiFeS-LDHs concentrations, chlorophyll content in ryegrass significantly decreased below CK levels at 650 and 800 mg/kg.

Figure 1C illustrates the effects of 2D NiFe-LDHs and 3D NiFeS-LDHs on H_2_O_2_ content in ryegrass. At 2D NiFe-LDHs concentration of 800 mg/kg and 3D NiFeS-LDHs concentrations of 200 and 800 mg/kg, H_2_O_2_ levels significantly increased, indicating notable oxidative stress in the high-concentration treatment groups. Compared with 2D NiFe-LDHs, 3D NiFeS-LDHs exerted a more pronounced impact on H_2_O_2_ levels in ryegrass.

Figure 1D shows the effects of 2D NiFe-LDHs and 3D NiFeS-LDHs on CAT activity in ryegrass. At all concentrations, CAT activity in the 3D NiFeS-LDHs group was significantly higher than that in the 2D NiFe-LDHs group and the CK group (*P* < 0.05). Figure 1E illustrates the impact of 2D NiFe-LDHs and 3D NiFeS-LDHs on SOD activity in ryegrass. SOD activity initially decreases and then increases with rising 2D NiFe-LDHs levels. Conversely, with increasing 3D NiFeS-LDHs concentrations, SOD activity initially increases and then decreases, with a turning point at 650 mg/kg. At 2D NiFe-LDHs concentrations of 200, 350, and 800 mg/kg, SOD activity significantly exceeds that observed in the CK group (*P* < 0.05). At concentrations ≤ 350 mg/kg, SOD activity in 2D NiFe-LDHs treatments is significantly higher than in 3D NiFeS-LDHs treatments (*P* < 0.05). However, at concentrations between 350 and 800 mg/kg, the trend reverses, with significantly lower SOD activity in 2D NiFe-LDHs treatments. Figure 1F illustrates that POD activity in ryegrass shows an increasing trend with rising NiFe-LDHs concentrations. A significant increase in POD activity is observed only at 800 mg/kg of 2D NiFe-LDHs (*P* < 0.05). In the 3D NiFeS-LDHs treatment group, a significant increase over the CK group occurs only at 200 mg/kg (*P* < 0.05). Significant differences are observed between the 2D NiFe-LDHs and 3D NiFeS-LDHs treatment groups at 350 and 800 mg/kg (*P* < 0.05).

#### The effect of LDHs on metal content of ryegrass

To further understand the impact mechanism of LDHs on ryegrass, the metal content in various plant parts was measured using ICP. As shown in Fig. S3, the Ni and Fe content in the leaves and roots of ryegrass treated with varying concentrations of 2D NiFe-LDHs and 3D NiFeS-LDHs is compared. The Ni concentration trend across the 2D NiFe-LDHs and 3D NiFeS-LDHs treatment groups shows consistent patterns, with Ni levels in the roots significantly higher than in the leaves. Ni content in the leaves initially increases and then decreases with rising soil concentrations of 2D NiFe-LDHs and 3D NiFeS-LDHs (Fig. S3A). At 650 mg/kg, leaf Ni content peaks, with no significant differences observed among all treatment groups. As the concentrations of 2D NiFe-LDHs and 3D NiFeS-LDHs increase, Ni content in the roots also rises (Fig. S3B). Although no significant differences are observed among different concentrations, root Ni content is consistently higher in the 3D NiFeS-LDHs group than in the 2D NiFe-LDHs group across all concentrations. In the 2D NiFe-LDHs treatment group, the Fe content in the leaf initially increased and then decreased as the concentration of NiFe-LDHs increased (Fig. S3C). No significant difference in the Fe content of the roots was observed between the 2D NiFe-LDHs and 3D NiFeS-LDHs treatment groups at varying concentrations (Fig. S3D).

### Effects of LDHs on soil

#### Effects of LDHs on soil physics and chemistry

**Fig. 2:**
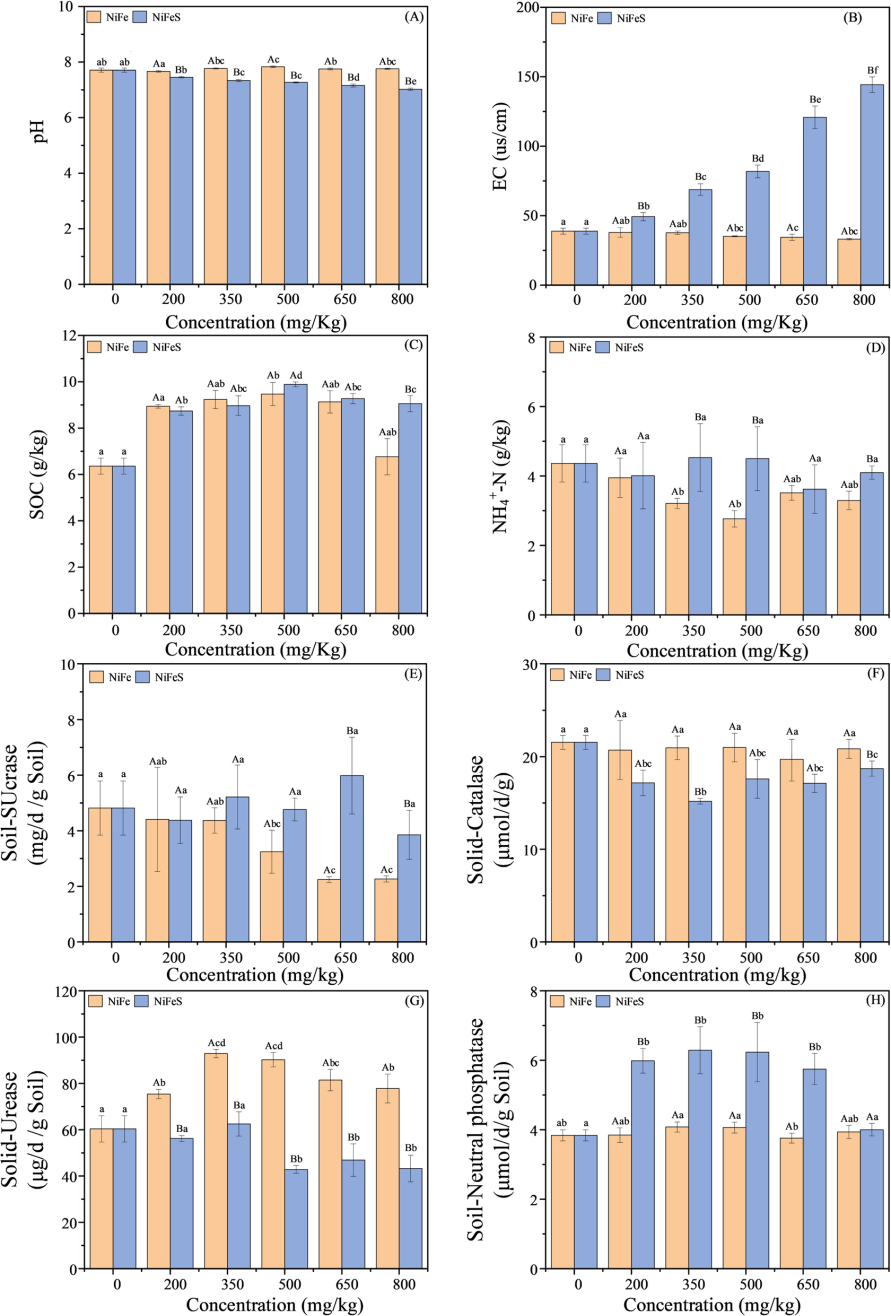
Illustrates the impact of LDH on soil pH (**A**), EC (**B**), SOC (**C**), NH_4_.^+^-N (**D**), S-SC (**E**), S-CAT (**F**), S-UE (**G**), and S-NP (**H**) activity. Lowercase letters on the bar chart denote significant differences between treatments, while uppercase letters indicate significant differences between 2D NiFe-LDHs and 3D NiFeS-LDHs (*P* < 0.05)

The physical and chemical properties of soil reflect its nutrient cycling efficiency and overall fertility. The results of the multifactor ANOVA (Fig. 2 and Table S3) indicate that 3D NiFeS-LDHs have a more pronounced effect on soil pH and EC compared to 2D NiFe-LDHs. Soil pH decreases significantly as 3D NiFeS-LDHs concentrations increase (Fig. 2A). In contrast, a different effect on EC was observed for NiFe-based LDH. In the 2D NiFe-LDHs groups, EC was reduced by 9.52%, 11.38%, and 14.92% at 500, 650, and 800 mg/kg, respectively, compared to the control (Fig. 2B). Conversely, EC in the 3D NiFeS-LDHs groups increased significantly compared to CK, with increases of 26.82%, 76.85%, 110.55%, 210.68%, and 271.13% at respective concentrations.

For TC, an increasing trend was observed in the 2D NiFe-LDHs group at 200–500 mg/kg concentrations. As the concentration of NiFe-LDH increased, a decline in TC and TP was noted. At concentrations of 350 and 500 mg/kg, the TC in the 2D NiFe-LDHs group was significantly higher than that in the corresponding 3D NiFeS-LDHs groups. In the 3D NiFeS-LDHs group, both TC and TP were generally lower than in the CK. At all concentrations, the total phosphorus (TP) in the 2D NiFe-LDHs group remained consistently higher than that in the 3D NiFeS-LDHs group, with no significant difference was observed.

The TN content in the soil of all NiFe-LDHs groups was significantly reduced by 32.39%, 28.71%, 31.55%, 32.00%, and 16.00% compared to the CK. Similarly, the TN in the 3D NiFeS-LDHs groups decreased significantly by 32.19%, 28.06%, 33.81%, 36.45%, and 33.29% compared to CK. The AP content in the 2D NiFe-LDHs groups gradually declined as the 2D NiFe-LDHs concentration increased, with significant reductions of 21.58% and 23.94% in the 650 mg/kg and 800 mg/kg compared to CK. The AP content in the five 3D NiFeS-LDHs treatment groups initially decreases and then increases as the 3D NiFeS-LDHs concentration rises. Compared to CK, AP in the 500 and 650 mg/kg 3D NiFeS-LDHs groups significantly decreases by 10.86% and 36.70%, respectively. Nevertheless, at 800 mg/kg, available phosphorus (AP) shows a significant increase of 8.82%.

Conversely, the SOC content in the 2D NiFe-LDHs treatment group initially increases and then decreases with rising NiFe concentrations (Fig. 2C). Compared to CK, soil organic carbon (SOC) increases significantly by 40.60%, 45.26%, 48.91%, and 43.62% in the 200, 350, 500, and 650 mg/kg 2D NiFe-LDHs groups, respectively. Similarly, in the NiFeS-LDHs group, SOC initially rises and then declines with concentration, showing significant increases of 37.43%, 41.06%, 55.53%, 45.84%, and 42.97% at 200, 350, 500, 650, and 800 mg/kg, respectively. Compared to CK, the NH_4_^+^-N content in the 500 mg/kg 2D NiFe-LDHs group decreased significantly by 30.29% (Fig. 2D). The 2D NiFe-LDHs groups at 350, 500, and 800 mg/kg showed significant reductions of 38.94%, 21.92%, and 19.58% compared to the 3D NiFeS-LDHs treatment groups at the same concentrations.

Analysis of the Ni and Fe content in the soil after 50 days of ryegrass cultivation revealed that Fe levels tended to be higher in the 2D NiFe-LDHs treatment groups compared to the 3D NiFeS-LDHs groups, but the differences were no significant difference observed. The yellow soil contains a substantial amount of Fe, while there is no significant difference in Fe content between the 2D NiFe-LDHs and 3D NiFeS-LDHs treatment groups and the untreated soil. A comparison of Ni content in soil treated with various concentrations of 2D NiFe-LDHs and 3D NiFeS-LDHs reveals that all 2D NiFe-LDHs treatment concentrations result in significantly higher Ni levels compared to the 3D NiFeS-LDHs treatments. Specifically, at concentrations of 200, 350, 500, 650, and 800 mg/kg, the Ni content in NiFe-treated soil increases by 20.75%, 24.72%, 28.57%, 47.10%, and 37.97%, respectively.

#### Effects of LDHs on soil enzyme

Soil enzyme activity indicates the level of biochemical reactions and nutrient cycling in soil, which quickly responds to changes in the soil environment. To examine the effects of 2D NiFe-LDHs and 3D NiFeS-LDHs on soil, we analyzed four key soil enzyme activities: S-UE, S-NP, S-CAT, and S-SC. As shown in Fig. 2E-H, NiFe-LDHs inhibits S-SC activity, promotes S-UE activity, and have no significant influence on S-CAT or S-NP. NiFeS-LDHs promote S-NP activity while inhibiting both S-SC and S-UE activity. Compared to the CK, S-SC activity in the 500, 650, and 800 mg/kg 2D NiFe treatment groups decreases significantly by 32.63%, 53.45%, and 52.97%, respectively. While NiFeS-LDHs have no significant effect on S-SC, its activity decreases progressively with increasing 3D NiFeS-LDHs concentration, indicating a dose-dependent trend. In contrast to 2D NiFe-LDHs, 3D NiFeS-LDHs at concentrations of 200, 350, 500, 650, and 800 mg/kg significantly inhibit S-CAT activity by 20.32%, 29.52%, 18.33%, 20.48%, and 13.16%, respectively. Conversely, 3D NiFeS-LDHs at concentrations of 200, 350, 500, and 650 mg/kg significantly enhance S-NP activity by 55.97%, 63.85%, 62.44%, and 49.74%. S-NP activity initially increases and then decreases as the concentration of 3D NiFeS-LDHs rises. 2D NiFe-LDHs and 3D NiFeS-LDHs treatments affect S-UE activity differently, though both exhibit a dose-dependent trend. Various concentrations of 3D NiFeS-LDHs significantly inhibit S-UE activity (6.86%−29.02%), while 2D NiFe-LDHs significantly enhance it (24.94%−53.91%). Changes in soil enzyme activity reflect alterations in the soil environment, which may indicate either a reduction or improvement in the ability of soil to decompose and transform organic materials.

#### Effects of LDHs on rhizosphere soil microbial community

The randomly selected sequences from CK, 2D NiFe-LDHs, and 3D NiFeS-LDHs soil samples and their corresponding ASVs were used to fit dilution curves. The ASV dilution curves for both soil bacterial (Fig. S4-5A) and fungal (Fig. S4-5B) samples in the control and treatment groups level off, indicating that the measured ASVs are approaching saturation. This suggests that the high-throughput sequencing data are reliable and accurately reflect the majority of bacterial and fungal community information in the samples. The Venn diagram shows the number of common and unique ASVs (operational taxonomic units) in a sample, visually displaying the similarity and overlap in ASV composition across soil samples. To compare the effects of 2D NiFe-LDHs and 3D NiFeS-LDHs on bacterial diversity in rhizosphere soil, ASV clustering was used to analyze gene sequences. Fig. S5 shows that 2D NiFe-LDHs share more species with the control group. Thus, 3D NiFeS-LDHs have a greater impact on bacterial and fungal species composition in rhizosphere soil than 2D NiFe-LDHs.

**Fig. 3:**
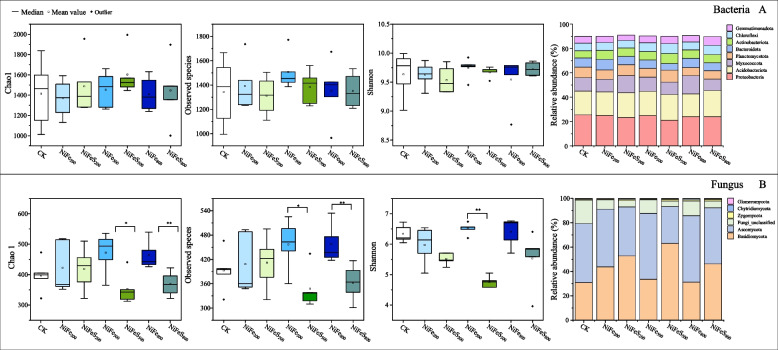
Alpha diversity of bacterial (**A**) and fungal (**B**) communities in soil treated with 2D NiFe-LDH and 3D NiFeS-LDHs. Note: Chao1 and Observed species indicate species richness, while Shannon index represents species diversity

2D NiFe-LDHs and 3D NiFeS-LDHs altered the alpha diversity of bacterial and fungal communities, affecting species richness (Chao1 index and Observed species) and diversity (Simpson and Shannon indices) (Fig. 3, Fig. S6, and Table S4). The α-diversity of bacterial communities initially increased and then declined with rising concentrations of 2D NiFe-LDHs and 3D NiFeS-LDHs, although no statistically significant differences were observed. At low concentrations, both 2D NiFe-LDHs and 3D NiFeS-LDHs slightly increased the Chao1 and Observed species indices of fungal community alpha diversity. At higher concentrations, 2D NiFe-LDHs and 3D NiFeS-LDHs exhibited different effects on fungal community α-diversity. In the 2D NiFe-LDHs group, α-diversity showed a moderate increase, but decreased in the 3D group. The 3D NiFeS-LDHs also had a stronger negative impact on the Simpson and Shannon indices.

Both 2D NiFe-LDHs and 3D NiFeS-LDHs were observed to modified microbial community diversity. Significant variations were detected in the relative abundances of the dominant microbial taxa across different NiFe-based LDH treatments, with the top 10 bacterial phyla (constituting approximately 97% of total sequence reads) and the top 6 fungal phyla (accounting for about 99.9% of total sequence reads) showing distinct compositional changes (Fig. 3 and Fig. S7, Tables S5-6). While some observed changes in alpha diversity did not reach statistical significance, the consistent shifts in beta diversity and taxon relative abundances (as shown in Fig. S7-S8) suggest community-level responses to LDH treatments. In contrast, treatment with 3D NiFeS-LDHs resulted in statistically significant decreases in fungal diversity indices at higher concentrations (Tables S7-S8 and Fig. S8).

**Fig. 4:**
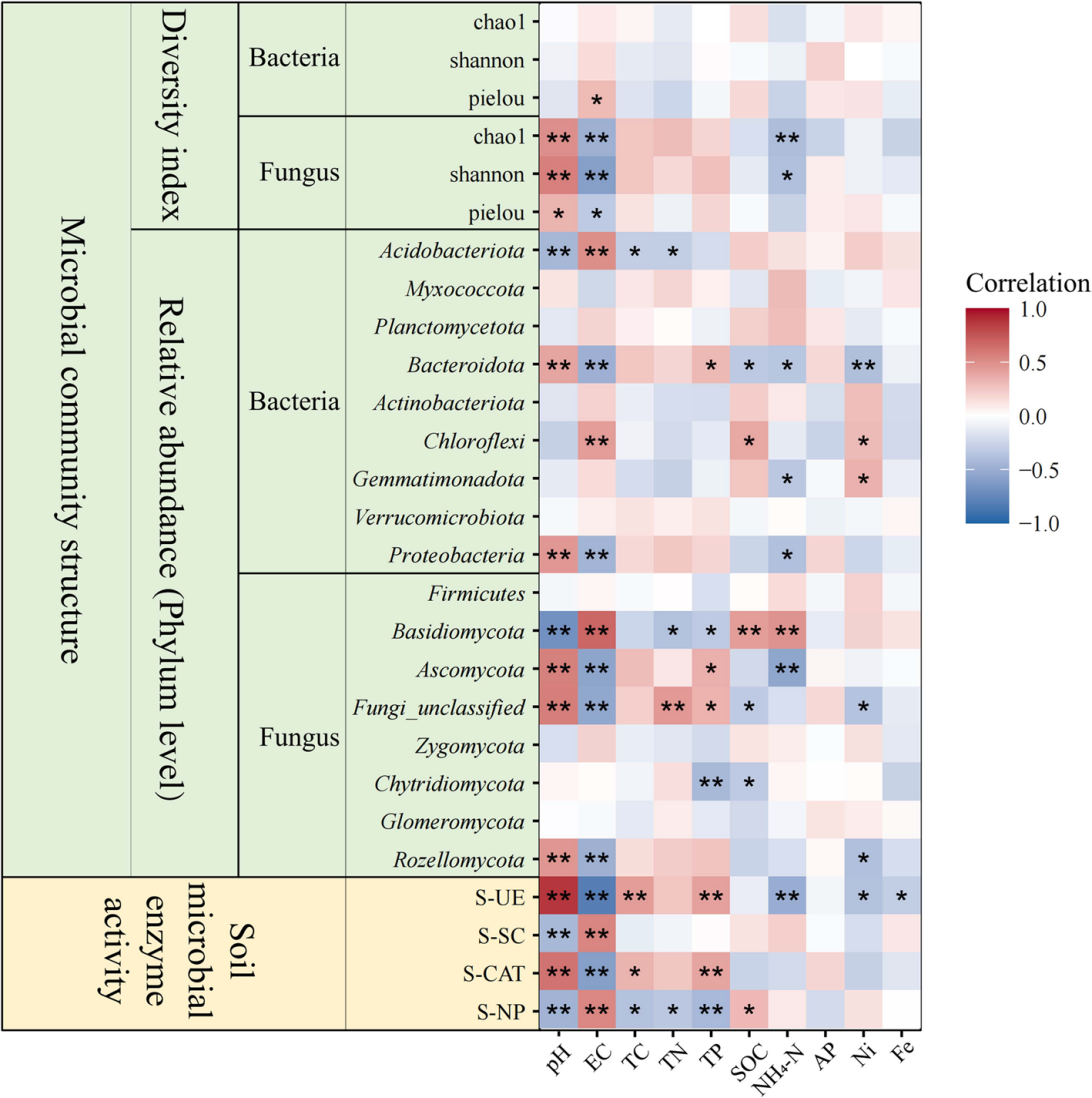
Pearson correlation heatmap between soil microbial alpha diversity, community structure (phylum level), and soil enzyme activities under NiFe-based LDH treatments. Red and blue indicate positive and negative correlations, respectively; Color depth represents the strength of correlation

A heatmap based on Pearson correlation analysis was generated to depict the relationships between soil properties, microbial characteristics, and soil enzyme activity (Fig. 4). The Pielou evenness index of bacterial communities is positively correlated with EC and negatively correlated with NH_4_^+^-N in the alpha diversity analysis. The Chao1 and Shannon indices of fungal communities are positively correlated with pH and negatively correlated with EC, NH_4_^+^-N and Fe. The Shannon index is also positively correlated with pH, but shows no significant correlation with EC, NH_4_^+^-N, or Fe.

**Fig. 5:**
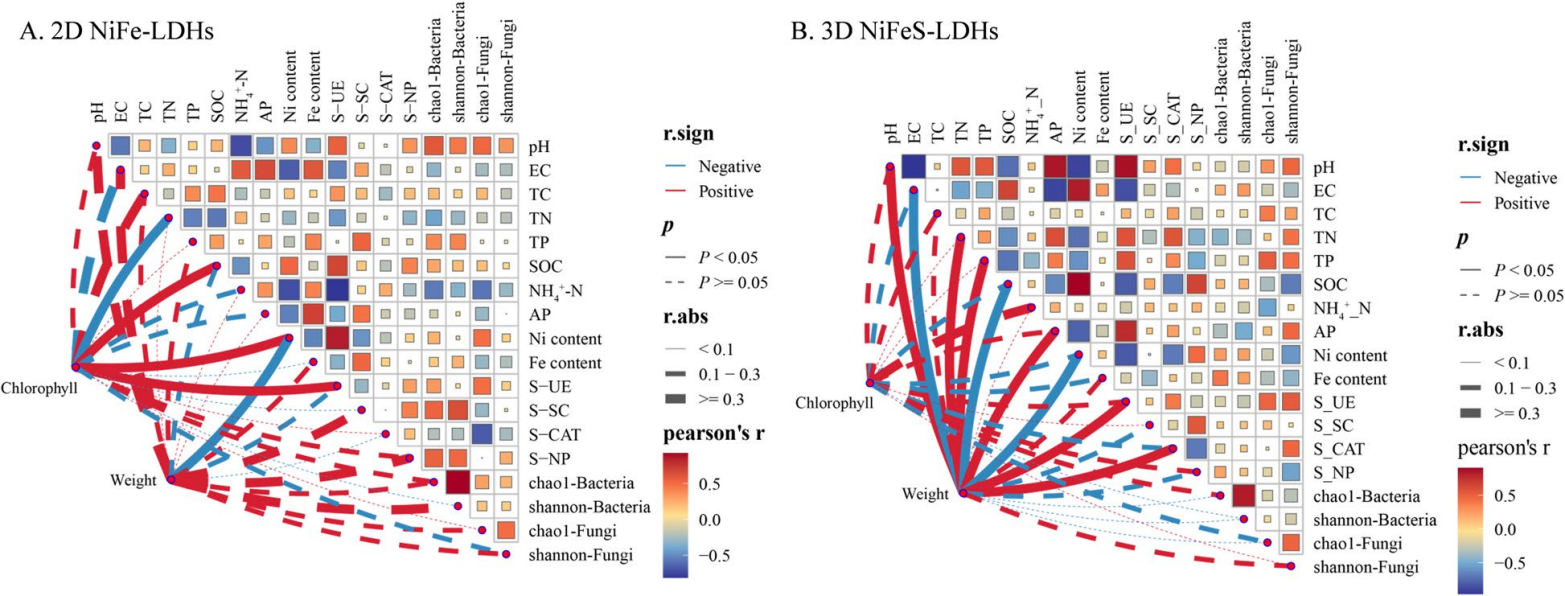
**A** Canonical correlation analysis between ryegrass growth and soil physicochemical properties, microbial diversity, and enzyme activities in the 2D NiFe-LDHs system, and (**B**) 3D NiFeS-LDHs system

Based on Pearson correlation analysis, this study identified key environmental factors significantly correlated with the fresh weight and soluble protein content of ryegrass. The canonical correlation analysis (Fig. 5) shows associations between soil properties, microbial diversity, enzyme activities and ryegrass growth, but does not imply causation.

**Fig. 6:**
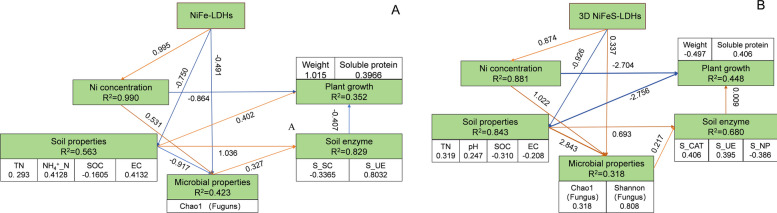
Partial least squares structural equation modeling (PLS-SEM) of the effects of (**A**) 2D NiFe-LDHs treatment and (**B**) 3D NiFeS-LDHs on ryegrass growth in soil environments

In the 2D NiFe-LDHs system (goodness-of-fit = 0.628), the residual NiFe-LDHs in the soil exerted a relatively weak overall effect on ryegrass growth through the ecosystem (total path coefficient = −0.368) (Fig. 6A). In contrast, within the 3D NiFeS-LDHs system (goodness-of-fit = 0.712), the overall negative impact of 3D NiFeS-LDHs on ryegrass growth via the soil ecosystem was significantly enhanced (total path coefficient = −1.071), indicating that the dimensional structure of the materials substantially influences their ecological effects (Fig. 6B). Specifically, soil physicochemical properties (total path coefficient = −1.933) and Ni concentration (path coefficient = −2.460) negatively regulated ryegrass growth, mainly due to soil acidification, salt stress, and nickel toxicity, whereas the microbial community positively influenced plant growth (path coefficient = 0.585), supporting its role in nutrient cycling and plant promotion. The weaker overall effect of 2D NiFe-LDHs (−0.368) compared to 3D NiFeS-LDHs (−1.071) reflects the greater environmental stress caused by the 3D material.

## Discussion

Physiological and biochemical indicators such as chlorophyll and H_2_O_2_ content are important for assessing the impact of LDHs on ryegrass growth. In our study, ryegrass exhibits physiological balance and stress responses to different concentrations of 3D NiFeS-LDHs. Low and moderate concentrations of 2D NiFe-LDHs (200–500 mg/kg) and 3D NiFeS-LDHs (200–350 mg/kg) stimulated chlorophyll synthesis, likely due to the beneficial effects of optimal Ni and Fe levels on chloroplast function. In particular, Ni played a crucial role in physiological processes such as urease activity and photosynthesis. However, at higher concentrations of 2D NiFe-LDHs and 3D NiFeS-LDHs, chlorophyll content decreased, possibly due to the suppression of chlorophyll synthesis caused by Ni or Fe accumulation, which disrupted chloroplast function (Lešková et al. 2020).

The intrinsic properties of ENMs, including morphology, particle size, surface charge, and hydrophilicity/hydrophobicity, influence plant development in different ways (Qiu et al. 2022). At NiFe-LDHs low and moderate concentrations, plants effectively alleviate oxidative stress induced by NiFe-LDHs through intrinsic regulatory mechanisms, maintaining cellular stability. At low concentrations, 3D NiFeS-LDHs cause greater H_2_O_2_ accumulation compared to 2D NiFe-LDHs, but, as the concentration increases, the difference in H_2_O_2_ levels between the two groups diminishes. This aligns with the observed pattern of certain nanomaterials promoting plant growth at low concentrations while inhibiting it at higher concentrations (Yang et al. 2021). Additionally, as a metal-based nanoparticle, NiFe-LDHs release metal ions such as Ni and Fe, which significantly impact plant growth (Tinwala and Wairkar 2019; He et al. 2019; Wang and Wang 2014). Ni is a trace element that, when used in moderation, promotes plant growth (Lešková et al. 2020), metabolism (Yadav et al. 2021), photosynthesis, and nutrient absorption. However, excessive amounts can generate ROS (H_2_O_2_), which adversely affects physiological and biochemical processes such as photosynthesis, transpiration (Aqeel et al. 2021), and mineral nutrition (Amjad et al. 2020), ultimately causing plant toxicity. At low concentrations, the contribution of dissolved metal ions from 3D NiFeS-LDHs to overall toxicity is less significant than that of released heavy metals. However, excessive Ni in the soil can impair iron absorption by plants, potentially leading to a deficiency of essential metals and reducing the biosynthesis of metal-dependent enzymes.

CAT, SOD, and POD are antioxidant enzymes that plants produce to maintain ROS homeostasis under stress. Our research shows that 2D NiFe-LDHs and 3D NiFeS-LDHs differently affect the activities of these enzymes. Low concentrations of 2D NiFe-LDHs enhance SOD activity in ryegrass, whereas high concentrations stimulate POD activity. In contrast, low to medium concentrations of 3D NiFeS-LDHs enhance CAT and POD activities, whereas high concentrations increase SOD activity. The differing oxidative stress effects of the two materials explain the variations in H_2_O_2_ content between the 2D NiFe-LDHs and 3D NiFeS-LDHs groups. 3D NiFeS-LDHs exhibited higher activity in soil and significantly influenced hydrogen peroxide concentration and antioxidant enzyme activity in ryegrass, effects closely linked to its three-dimensional structure. It induced a more pronounced oxidative stress response, resulting in increased production of H_2_O_2_ and other ROS in plant cells, thereby markedly enhancing antioxidant enzyme activity. In contrast, 2D NiFe-LDHs, owing to its stable structure and lower oxidative reactivity, exerted a milder impact on plant oxidative stress, leading to more stable changes in enzyme activity. This indicates its ability to exert a mild regulatory effect within a specific concentration range.

Differences in LDHs types resulted in significant variations in the oxidative stress response of organisms (Yu et al. 2023; Wang et al. 2023). This difference in toxicity is attributed to the particle dispersion stability and metal composition of the LDHs (Yu et al. 2023). At low concentrations, the overall toxicity of 3D NiFeS-LDHs is more influenced by its particle morphology than by dissolved metal ions, and its toxicity is higher than that of 2D NiFe-LDHs. Analysis of heavy metal content in leaves and roots reveals that Ni concentration in ryegrass leaves treated with low to medium concentrations of 2D NiFe-LDHs is slightly higher than in those treated with 3D NiFeS-LDHs. Ni is essential for promoting plant development and growth, particularly at low concentrations (0.05–10 mg/kg dry weight). At low concentrations, 2D NiFe-LDHs exhibits lower toxicity than 3D NiFeS-LDHs. With increasing concentrations, the toxic effects of 2D NiFe-LDHs and 3D NiFeS-LDHs on ryegrass converge. At high concentrations, 3D NiFeS-LDHs exhibits reduced stability and excessive Ni release, leading to heightened oxidative stress responses in ryegrass that may exceed acceptable levels.

The addition of two types of NiFe-based LDHs to the soil changed the soil environment, and there were significant differences between the two treatments. Soil pH served as a key indicator of geochemical properties, critically influencing nutrient availability and the activity of toxic metals (Wang et al. 2022). The significant soil pH decrease caused by 3D NiFeS-LDHs results from several combined processes. First, oxidation of the sulfide component in aerobic soil produces sulfate and H^+^ (Schippers 2004). Ni sulfides undergo similar oxidation, releasing Ni^2+^, SO_4_^2−^, and H^+^. Second, the released Ni^2+^ and Fe^3+^ hydrolyze, consuming OH- and releasing more H^+^. Third, under acidic conditions, iron-oxidizing microbes (e.g., Acidithiobacillus ferrooxidans) oxidize Fe^2+^ to Fe^3+^, and the regenerated Fe^3+^ further oxidizes remaining sulfides, creating a cycle that continuously generates H^+^ (Newsome and Falagán 2021). Together, these processes drive soil acidification (Fig. 2A). Correlation analysis demonstrated that soil pH and EC values were significantly correlated with the relative abundance of specific microbial taxa at both phylum and genus levels (Fig. S7). Due to its 2D layered structure and low solubility, 2D NiFe-LDHs does not readily release large amounts of electrolytes into the soil, causing EC to gradually decrease with increasing concentration (Rosa et al. 2019). This may be attributed to the adsorption or immobilization of certain metal ions, reducing their concentration in the soil solution. In contrast, sulfides are highly reactive chemical components, particularly in aerobic environments, where they are easily oxidized to form soluble electrolytes such as sulfate ions. The 3D structure of NiFeS-LDHs maximizes exposed reaction sites, promoting ion release (Chala et al. 2020). Thus, increasing concentrations lead to a rapid rise in soluble ions (e.g., S, Ni, and Fe ions) in the soil, significantly enhancing EC. Additionally, compared with 3D NiFeS-LDHs, 2D NiFe-LDHs exhibits a stabilizing effect on the levels of TC, TP, and Fe in the soil. The contrasting AP trends reflect the different properties of the two LDHs. 2D NiFe-LDHs steadily adsorb phosphate via anion exchange, causing a gradual AP decline. For 3D NiFeS-LDHs, strong adsorption at low-moderate concentrations is reversed at 800 mg/kg, where soil acidification (pH drop) and increased EC promote phosphate desorption and mineral dissolution, leading to a net AP increase.

Soil enzyme activity is a key indicator of pollutant decomposition, soil substance transformation, and environmental changes. In our study, 2D NiFe-LDHs inhibited S-SC activity and promoted S-UE activity, while having no significant effect on S-NP or S-CAT activity. Medium concentrations of 3D NiFeS-LDHs promoted S-NP activity but inhibited S-CAT and S-UE, with no significant impact on S-SC activity. These findings align with previous reports on ENMs, showing that the same type of ENM can influence different soil enzyme activities in diverse ways (Asadishad et al. 2018). Additionally, nanoparticle effects on soil enzyme activity depend on factors such as shape and structure (Moon et al. 2019), particle size, chemical composition, exposure time (Zhai et al. 2021), and soil type (Jośko et al. 2014). Structural differences among ENMs generally influence their impacts on soil enzyme activity (Zhai et al. 2016), as supported by our findings.

Research indicates that LDHs influence soil enzyme activity both directly and indirectly by altering the soil environment (Li et al. 2024). Differences in the effects of 2D NiFe-LDHs and 3D NiFeS-LDHs on soil enzyme activity observed in our study were likely due to variations in nutrient fixation mechanisms. Specifically, 2D NiFe-LDHs demonstrated a stronger fixation effect on total carbon (TC) and total phosphorus (TP) compared to 3D NiFeS-LDHs, which impeded the decomposition and transformation of these nutrients. This result corresponds with the higher TC and TP contents observed in soils treated with 2D NiFe-LDHs. Soil pH and EC significantly influenced enzyme responses, with notable differences between treatments corroborating our correlation analysis. These changes directly affected the activity of the four soil enzymes examined in this study.

Soil microorganisms are crucial indicators for nutrient cycling and assessing pollutant impacts on soil (Yamini et al. 2023). Among heterotrophic microorganisms, bacterial populations, constituting approximately 15% of the total microbial community (Govindasamy et al. 2011), have been reported to directly or indirectly promote plant growth (Zegzouti et al. 2019). The presence of metal-based ENMs significantly altered the structure of soil bacterial and fungal communities, impacting their diversity and richness (Wu et al. 2021). Our findings confirmed that NiFe-based LDHs affected the relative abundance of dominant microorganisms at both the phylum and genus levels (Fig. 6 and S6, Tables S7-8, and S9), with changes dependent on the type and concentration of LDHs. Moderate concentrations of 2D NiFe-LDHs increased the abundance of *Bryobacter*, *Flavisolibacter*, *Rhodoplanes*, *Cryptococcus* and *Fusarium*. These taxa were associated with nitrate reduction (He et al. 2023), plant growth promotion (Mehmood et al. 2022), nitrogen conversion and denitrification (Ye et al. 2024), and stress resistance (Cecilia Mestre et al. 2011). The relative abundance of *Anaeromyxobacter*, associated with nitrogen conversion (Yuan et al. 2017), increased moderate concentrations but declined at higher levels of 3D NiFeS-LDHs. The microbial diversity in the 3D NiFeS-LDH treatment group was lower compared to CK and 2D NiFe-LDHs. Increased fungal diversity has been linked to improved soil health, crop productivity, and agricultural sustainability. The effects of the two LDHs materials on plants were mediated both directly, through changes in soil physicochemical properties, and indirectly, by altering the dominant microbial communities. Soil microorganisms exhibited distinct responses to varying dosages of 2D NiFe-LDHs and 3D NiFeS-LDHs.

One mechanism by which ENMs affect microbes is by providing attachment sites for specific microbial communities in the soil due to their surface properties (Hook et al. 2012). This adhesion may influence the attachment of specific microorganisms to solid surfaces and extracellular polymeric substances, as well as bacterial colonization, ultimately altering the relative abundance of dominant soil microbial populations (Ameen et al. 2021). Additionally, LDHs impacts microbial communities directly by modifying physiological and biochemical processes, and indirectly by altering soil characteristics (Li et al. 2024). Changes in the relative abundance of soil microorganisms suggest that 2D NiFe-LDHs and 3D NiFeS-LDHs exert selective effects on bacterial and fungal proliferation, thereby supporting normal soil function and fertility through changes in microbial community diversity. Correlation analysis indicates that different types of LDHs influence soil physicochemical properties (Fig. 4 and Fig. S7), particularly pH and EC, in distinct ways, leading to varying impacts on microbial diversity. A significant correlation was observed between soil nutrients and the relative abundance of certain dominant bacterial genera (Fig. S7). For instance, NH^4+^-N positively correlated with *Vicinamibacteraceae*_*unclassified*, *Rhodoplanes*, *Gemmatimonadota*_*nclassified*, *Subgroup-7_unclassified*, *Halimium*, Pirellulaceae unclassified, and *Firmicutes_unclassified*, but negatively correlated with *Anaeromyxobacter*, *Vicinamibacterales_unclassified*, and *Flavisolibacter*. These bacterial taxa were likely involved in nitrogen cycling. The effect of LDHs on the rhizosphere microenvironment may further influence plant growth (Sillen et al. 2015). Notably, the type of LDHs appeared to determine its biological effects.

In conclusion, our study demonstrated that the type and dosage of NiFe-based LDHs influenced ryegrass growth, soil geochemistry, and enzyme activity. NiFe-based LDHs directly impacted soil geochemistry and significantly altered the structure of soil fungal communities. The shifts in microbial communities resulted in the enrichment of specific taxa with potential degradation capabilities and suggesting potential shifts in soil ecological functions, which were further supported by changes in soil enzyme activities and their stoichiometric ratios. Compared to 2D NiFe LDHs, 3D NiFeS-LDHs had a more pronounced biological impact on the ryegrass-soil ecosystem, particularly on nitrogen cycling processes. However, the biological effects of metal-based NiFe-LDHs are highly complex. Material properties (e.g., structure, composition, surface charge), exposure duration, soil type, microbial cell wall characteristics, and plant species significantly influenced their ecological toxicity. Further case studies are essential to elucidate the biological effects of NiFe-based LDHs, which is crucial for evaluating their potential sustainable applications.

## Conclusion

This study examined the ryegrass-soil system to assess the effects of varying concentrations of NiFe-based LDHs, an innovative engineered nanomaterial with distinct morphologies and structures, on ryegrass growth, soil geochemical, enzyme activity, and microbial community composition (bacterial and fungal). Compared with previous LDH toxicity studies focusing on aquatic algae, this work provides evidence in a terrestrial plant-soil system. The results indicated that ryegrass growth, soil properties, and enzyme activities were influenced by both the type and dosage of NiFe-based LDHs. Both materials induced oxidative stress, with 3D NiFeS-LDHs more effectively stimulating CAT and POD activity of ryegrass, although enzyme activity declined at higher concentrations. In contrast, 2D NiFe-LDHs stimulated SOD and POD activity. In soil systems, 3D NiFeS-LDHs significantly reduced soil pH while increasing EC, SOC, and NH_4_^+^-N levels, due to its larger surface area and complex structure. Similarly, 2D NiFe-LDHs significantly raised AP and SOC contents, stimulating S-NP activity but inhibiting sucrase at medium to high concentrations. 3D NiFeS-LDHs enhanced S-NP activity while suppressing soil catalase and urease activity. Both NiFe-based LDHs altered the diversity of soil bacterial and fungal communities, with 3D NiFeS-LDHs exerting a stronger inhibitory effect on fungal diversity at higher concentrations. These materials also reshaped microbial community composition and soil enzyme activities, with enzyme stoichiometric ratios indicating shifts in soil metabolic function. These effects depended on the type and dosage of LDHs. The observed changes in ryegrass growth, soil geochemistry, and enzyme activity were attributed to the direct impact of LDHs on soil physicochemical properties and its influence on microbial community composition and function. The study concluded: (1) Both 2D and 3D NiFe-based LDHs affected ryegrass growth, soil microbial communities, and geochemical properties, with the impacts varying by type and dosage; (2) 3D NiFeS-LDHs exhibited a stronger biological effect on the ryegrass-soil ecosystem, particularly on nitrogen cycling processes. The actual field toxicity of NiFe-based LDHs requires future validation of causal pathways via multi-season trials, ionic/bulk controls, and molecular studies; nonetheless, this study provides a basis for assessing their acute soil toxicity.

## Supplementary Information

Supplementary Material 1.

## Data Availability

All data generated or analyzed during this study are included in this published article [and its supplementary information fles].
