# Supplementary material for: Bio-effects of engineering nanomaterials NiFe-based LDHs on ryegrass-soil system
Source: Adv Biotechnol (Singap). 2026 Jun 24;4(3):24. doi: 10.1007/s44307-026-00114-x (PMC13291365; doi:10.1007/s44307-026-00114-x)
Supplement: Supplementary file 1 — Supplementary Material 1. [file 44307_2026_114_MOESM1_ESM.docx]

**Supplementary Information**

**Bio-effects of Engineering Nanomaterials NiFe-based LDHs on Ryegrass-Soil System**

Haocheng Xu^a,b^, Xiaoqian Jiang^a,b^, Chuntao He^a,b^, Yutao Peng^a,b^, Guorong Xin^a,b,*^, Xiaoyun Li^a,b,*^

^a^ State Key Laboratory of Biocontrol, Guangdong Provincial Key Laboratory of Plant Stress Biology, School of Agriculture and Biotechnology, Sun Yat-sen University, Shenzhen, Guangdong, 518107, PR China

^b^ School of Agriculture and Biotechnology, Sun Yat-sen University, Shenzhen, Guangdong, 518107, PR China

**Text S1. Plant culture**

The sowing rate was 2.7 g/m², using a broad-leaved variety of Italian ryegrass adapted to local conditions, with 4 replicates per treatment. Before sowing ryegrass, the seeds were disinfected with a 5% HClO_3_ solution. The seeds were soaked in the disinfectant for half an hour and then rinsed thoroughly with ultrapure water. The ryegrass was harvested 50 days after indoor cultivation (12 h of light at 20 °C), with equal amounts of water added every three days.

**Text S2. Plant harvesting and soil sample collection**

All blades were cut into small pieces, mixed evenly, frozen in liquid nitrogen, and immediately ground. These samples were stored at -80°C for further analysis. Soil samples included rhizosphere and non-rhizosphere soil. Non-rhizosphere soil referred to soil around the roots that was not tightly bound. Rhizosphere soil was collected from soil closely bound to the roots (Guan et al. 2020). The collected non-rhizosphere soil was air-dried, ground, sieved, and stored at 4 °C for further analysis of basic physicochemical properties and enzyme activity. The collected rhizosphere soil was mixed and stored at -80 °C for microbial community analysis.

**Text S3. Growth physiological indicators of ryegrass**

**Soluble protein**: Accurately weighed 1.000 g of plants were ground into a pulp and centrifuged at 4000 r/min for 20 min. The supernatant was diluted to 50 ml with distilled water to obtain the extraction solution. 5 mL of Coomassie Brilliant Blue G-250 reagent was mixed with 0.1 mL of the extraction solution, left to stand for 2 min, and the protein content was measured at 595 nm.

**H_2_O_2_** **content:** Weighed 1.000 g of plants were ground into a slurry with 1 mL of pre-cooled acetone at 4 °C. The homogenate was centrifuged at 3000 r/min for 10 min, and the supernatant was collected. 0.1 mL of 5% titanium sulfate and 0.2 mL of concentrated ammonia water were added, allowed to precipitate, and then centrifuged at 3000 r/min for 10 min. The supernatant was discarded, and the precipitate was washed multiple times with acetone. The washed precipitate was dissolved in 2 mol/L H_2_SO_4_ and made up to 10 mL. The H_2_O_2_ content was measured at a wavelength of 415 nm.

**Determination of Peroxidase (POD) Activity:** 0.4 g of fresh leaves were ground into a slurry with 8 mL of pre-cooled 50 mmol/L phosphate (PB) buffer (pH 7.8), then centrifuged at 10,000 r/min for 15 min. The supernatant was diluted to 10 mL with 50 mmol/L PB and stored at 4 °C. To 0.2 mL of enzyme extraction solution, 5 mmol/L PB buffer, 2% H_2_O_2_ solution, and 5 mmol/L guaiacol solution were added sequentially and incubated at 37 °C for 5 min. The absorbance of the reaction system was measured at a wavelength of 534 nm.

**Determination of superoxide dismutase (SOD) activity:** 0.5 g of fresh leaves were ground in 1 mL of PB buffer under ice bath conditions. The mixture was then adjusted to a total volume of 5 mL with PB buffer. After centrifuging 2-3 mL at 10,000 r/min for 10 min, the supernatant was obtained as a crude enzyme extract. To 50 µL of the crude enzyme extract, 0.5 mmol/L PB buffer, 130 mmol/L DL-methionine solution, 750 mmol/L nitro-tetrazolium chloride blue solution, 750 mmol/L disodium ethylenediaminetetraacetate, 20 mmol/L riboflavin, and water were sequentially added and thoroughly mixed. The absorbance of the reaction system was measured at a wavelength of 560 nm.

**Determination of catalase (CAT) activity:** 0.5 g of fresh leaves were homogenized into a slurry by adding 3 mL of PB buffer at pH. The mixture was then adjusted to a volume of 10 mL and allowed to stand at 5 °C for 10 min. After centrifuging the supernatant at 4000 r/min for 15 min, it was collected and reacted with 0.2 mol/L PB solution at pH 7.8 and H_2_O_2_. The absorbance value of the reaction system was measured at 240 nm.

**Text S4. Soil enzyme activities**

Soil enzyme activities: Measured soil sucrase (S-SC) activity by adding 15 µL of toluene to 0.1 g of air-dried soil in a centrifuge tube. Shook and mixed well to moisten the soil sample completely, then placed it in a water bath at 37 ℃ for 15 min. Used the soil sucrase assay kit (SSC-2-Y) from Comin Biotechnology for determination. Measured soil catalase activity (S-CAT) by weighing 0.1 g of air-dried soil and using the Comin Biotechnology Catalase Kit (SCAT-2-Y). Recorded the results. Measured soil phosphatase (S-NP) activity by adding 50 µL of toluene to 0.1 g of air-dried soil in a centrifuge tube. Shook and mixed thoroughly, moistened the soil completely, and incubated at 37 °C for 24 h. Used the Comin Biotechnology Soil Phosphatase Assay Kit (SNP-1-W) for determination. Measured soil urease (S-UE) activity by adding 125 µL of toluene to 0.25 g of air-dried soil in a centrifuge tube. Shook and mixed thoroughly, moistened the soil completely, and let it stand at room temperature for 15 min. Used the Comin Biotechnology Soil Urease Test Kit (SUE-2-Y) for determination.

**Text S5. Material and method description of 16SrDNA sequencing**

**DNA extractions:** DNA from different samples was extracted using the CTAB according to manufacturer’s instructions. The reagent which was designed to uncover DNA from trace amounts of sample has been shown to be effective for the preparation of DNA of most bacteria. Nuclearfree water was used for blank. The total DNA was eluted in 50 μL of Elution buffer and storedat -80 °C until measurement in the PCR by LCBio TechnologyCo., Ltd, Hang Zhou, Zhejiang Province, China.

**PCR amplification and 16S rDNA sequencing:** The 5' ends of the primers were tagged with specific barcods per sample and sequencing universal primers.PCR amplification was performed in a total volume of 25 μL reaction mixture containing 25 ng of template DNA, 12.5 μL PCR Premix, 2.5 μL of each primer, and PCR-grade water to adjust the volume. The PCR conditions to amplify the prokaryotic 16S fragments consisted of an initial denaturation at 98 ℃ for 30 seconds; 32cycles of denaturation at 98 ℃ for 10 seconds, annealing at 54℃ for 30 seconds, and extension at 72 ℃ for 45 seconds; and then final extension at 72 ℃ for 10 minutes. The PCR products were confirmed with 2% agarose gel electrophoresis. Throughout the DNA extraction process, ultrapure water, instead of a sample solution, was used to exclude the possibility of false-positive PCR results as a negative control. The PCR products were purifyied by AMPure XT beads (Beckman Coulter Genomics, Danvers, MA, USA) and quantified by Qubit (Invitrogen, USA). The amplicon pools were prepared for sequencing and the size and quantity of the amplicon library were assessed on Agilent 2100 Bioanalyzer (Agilent, USA) and with the Library Quantification Kit for Illumina (Kapa Biosciences, Woburn, MA, USA), respectively. The libraries were sequenced on NovaSeq PE250 platform. 16S rDNA sequencing results are presented in Table S1.

**Data analysis:** Samples were sequenced on an Illumina NovaSeq platform according to the manufacturer's recommendations, provided by LC-Bio. Paired-end reads were assigned to samples based on their unique barcode and truncated by cutting off the barcode and primer sequence. Paired-end reads were merged using FLASH. Quality filtering on the raw reads was performed under specific filtering conditions to obtain the high-quality clean tags according to the fqtrim(v0.94). Chimeric sequences were filtered using Vsearch software (v2.3.4). After dereplication using DADA2, we obtained feature table and feature sequence.Alpha diversity and beta diversity were calculated by normalized to the same sequences randomly.Then according to SILVA (release 138) classifier, feature abundance was normalized using relative abundance of each sample. Alpha diversity is applied in analyzing complexity of species diversity for a sample through 5 indices, including Chao1, observed species, Goods coverage, Shannon, Simpson, and all these indices in our samples were calculated with QIIME2. Beta diversity was calculated by QIIME2, the graphs were drawn by R package.Blast was used for sequence alignment, and the feature sequences were annotated with SILVA database for each representative sequence.Other diagrams were implemented using the R package (v3.5.2)

Fig. S1 The XRD image of 2D NiFe-LDHs (A) and 3D NiFeS-LDHs (B), Data adapted from our previous work (Xu et al., 2025).

Fig. S2 The SEM image of 2D NiFe-LDHs (A) and 3D NiFeS-LDHs (B), Data adapted from our previous work (Xu et al., 2025).

Fig. S3 The effect of LDH on Ni and Fe content in different parts of ryegrass. Lowercase letters on the bar chart denote significant differences between treatments, while uppercase letters indicate significant differences between 2D NiFe-LDHs and 3D NiFeS-LDHs (*P*<0.05).

Fig. S4 bacterial (a) and Fungal (b) observed ASVs rarefactions of soil samples based on the sequencing depth

Fig. S5 Effects of LDHs on soil bacterial diversity: Venn diagram of species distribution based on ASV.

Fig. S6 Simpson's alpha diversity index for bacterial (A) and fungal (B) communities in soil treated with 2D NiFe-LDHs and 3D NiFeS-LDHs.

Fig. S7 The heatmap showing the relative abundance of the top 50 bacterial genera (a) and top 50 fungus genera in the soil exposed to 2D NiFe-LDHs and 3D NiFeS-LDHs. The blue color means positive correlation, and red color means negative correlation

Fig. S8 Correlation between soil properties and the top 30 bacterial (A) and fungal genera. Note: Red and blue for positive and negative correlation, respectively. Color depth represents the degree of relationship.

**Table S1** Characterization results of NiFe-based LDHs

|  | Specific surface area  (m^2^/g) | Pore size  (nm) | Pore volume  (cm^3^/g) | Electrical conductivity  (mS/cm^2^) | Zeta potential  (mS/cm) |
| --- | --- | --- | --- | --- | --- |
| NiFe-LDHs | 43.61 | 13.23 | 0.19 | 0.16 | 23.16 |
| NiFeS-LDHs | 114.07 | 9.56 | 0.35 | 0.62 | 4.35 |

**Table S2** PCR amplification and 16S rDNA sequencing

| Region | Primers |
| --- | --- |
| V3-V4 (Logue, et al. 2016) | 341F(5’-CCTACGGGNGGCWGCAG-3’)  805R(5’-GACTACHVGGGTATCTAATCC-3’) |
| Archae (Ken, et al. 2000) | F(5’-GYGCASCAGKCGMGAW-3’)  R(5’-GGACTACHVGGGTWTCTAAT-3’) |
| V4 (Walters, etal. 2015) | 515F(5’-GTGYCAGCMGCCGCGGTAA-3’)  806R(5’GGACTACHVGGGTWTCTAAT-3’) |
| V4-V5 | F(5’-GTGCCAAGCMGCCGCGG-3’)  R(5’-CCGTCAATTCMTTTRAGTTT-3’) |

**Table S3** Soil Physical and Chemical Properties of NiFe and NiFeS Treatment Groups

| Materials | Concentration | pH | EC  (μs/cm) | TC (g/kg) | TP (g/kg) | TN (g/kg) | | AP (ug/kg) | SOC (g/kg) | NH4+-N (mg/kg) | Fe (g/kg) | Ni (g/kg) |
| --- | --- | --- | --- | --- | --- | --- | --- | --- | --- | --- | --- | --- |
| CK | 0 | 7.71±0.08 ab | 38.86±2.15 a | 13.55±0.51 a | 0.31±0.020 Aa | 1.55±0.09 a | 7.03±0.30 a | | 6.36±0.35 a | 3.97±0.90 a | 11.33±0.28 a | 0.004±0.001 a |
| NiFe | 200 | 7.66±0.03 Aa | 37.876±3.44 Aab | 14.04±0.83 Aa | 0.315±0.010 Aa | 1.05±0.04 Ab | 7.07±0.63 Aa | | 8.94±0.08 Ab | 3.95±0.57 Aa | 10.92±0.44 Aa | 0.064±0.003 Ab |
|  | 350 | 7.77±0.02 Abc | 37.75±1.19 Aab | 14.12±0.07 Aa | 0.31±0.020 Aa | 1.11±0.13 Ab | 6.48±0.99 Aa | | 9.24±0.40 Ab | 3.21±0.15 Aab | 12.06±1.18 Aa | 0.111±0.006 Ac |
|  | 500 | 7.83±0.03 Ac | 35.18±0.37 Abc | 14.39±0.89 Aa | 0.31±0.009 Aa | 1.06±0.05 Ab | 6.67±0.30 Aa | | 9.47±0.50 Ab | 2.77±0.24 Ab | 11.34±0.55 Aa | 0.153±0.004 Ad |
|  | 650 | 7.75±0.03 Ab | 34.45±2.26 Ac | 13.57±0.85 Aa | 0.30±0.006 Aa | 1.05±0.06 Ab | 5.52±0.29 Ab | | 9.13±0.48 Ab | 3.51±0.21 Aab | 11.34±0.74 Aa | 0.203±0.006 Ae |
|  | 800 | 7.76±0.02 Abc | 33.08±0.58 Ac | 13.48±0.64 Aa | 0.29±0.030 Aa | 1.30±0.14 Ac | 5.35±0.34 Ab | | 6.77±0.78 Aa | 3.30±0.27 Aab | 11.88±2.26 Aa | 0.218±0.011 Af |
| NiFeS | 200 | 7.45±0.03 Bb | 49.30±2.97 Bb | 13.56±0.20 Aa | 0.29±0.026 Aa | 1.05±0.02 Abc | 6.77±0.14 Aab | | 8.74±0.18 Ab | 4.01±0.96 Aa | 11.46±0.27 Aa | 0.053±0.003 Bb |
|  | 350 | 7.34±0.04 Bc | 68.75±4.15 Bc | 12.94±0.30 Bb | 0.29±0.041 Aa | 1.12±0.11 Ab | 6.48±0.45 Aab | | 8.97±0.42 Abc | 4.53±0.98 Ba | 10.17±0.77 Ab | 0.089±0.004 Bc |
|  | 500 | 7.27±0.02 Bc | 81.85±4.57 Bd | 13.31±0.35 Bab | 0.28±0.007 Aa | 1.03±0.03 Abc | 6.27±0.36 Ab | | 9.89±0.19 Ad | 4.50±0.92 Ba | 10.89±0.54 Aab | 0.119±0.002 Bd |
|  | 650 | 7.16±0.05 Bd | 120.78±8.15 Be | 12.93±0.33 Ab | 0.29±0.013 Aa | 0.99±0.03 Ac | 4.45±0.30 Bc | | 9.27±0.22 Abc | 3.62±0.670 Aa | 11.57±0.63 Aa | 0.138±0.001 Be |
|  | 800 | 7.02±0.04 Be | 144.28±5.62 Bf | 13.63±0.18 Aa | 0.28±0.008 Aa | 1.03±0.01 Bbc | 7.65±0.18 Bd | | 9.06±0.35 Bc | 4.10±0.19 Ba | 10.91±0.80 Aab | 0.158±0.002 Bf |

**Table S4** Comparison of Alpha Diversity Differences in fungus Communities.

| alpha diversity of fungus communities | group1 | group2 | *P* | *P*.significance |
| --- | --- | --- | --- | --- |
| observed species | NiFeS_500_ | NiFe_500_ | 0.02 | * |
|  | NiFeS_500_ | NiFe_650_ | 0.03 | * |
|  | NiFeS_500_ | NiFe_800_ | 0.03 | * |
|  | NiFeS_800_ | NiFe_800_ | 0.01 | ** |
| shannon | NiFeS_200_ | NiFeS_500_ | 0.01 | ** |
|  | NiFeS_200_ | CK | 0.01 | ** |
|  | NiFeS_350_ | NiFe_350_ | 0.01 | ** |
|  | NiFeS_350_ | CK | 0.01 | ** |
|  | NiFeS_500_ | NiFe_500_ | 0.01 | ** |
| simpson | NiFeS_200_ | NiFeS_500_ | 0.01 | ** |
|  | NiFeS_200_ | NiFe_200_ | 0.01 | ** |
|  | NiFeS_200_ | CK | 0.01 | ** |
|  | NiFeS_350_ | NiFeS_500_ | 0.02 | * |
|  | NiFeS_350_ | NiFe_350_ | 0.01 | ** |
|  | NiFeS_350_ | CK | 0.01 | ** |
|  | NiFeS_500_ | NiFe_500_ | 0.01 | ** |
|  | NiFeS_500_ | CK | 0.01 | ** |
|  | NiFeS_650_ | NiFe_650_ | 0.01 | ** |
|  | NiFeS_650_ | CK | 0.01 | ** |
|  | NiFeS_800_ | CK | 0.02 | * |
|  | NiFeS_350_ | NiFeS_500_ | 0.01 | ** |
| chao1 | NiFeS_500_ | NiFe_500_ | 0.02 | * |
|  | NiFeS_800_ | NiFe_800_ | 0.01 | ** |

**Table S5** Relative abundance (%) of the top 10 bacterial phyla.

|  | Top 10 bacterial phyla | | | | | | | | | |
| --- | --- | --- | --- | --- | --- | --- | --- | --- | --- | --- |
|  | Proteobacteria | Acidobacteriota | Myxococcota | Planctomycetota | Bacteroidota | Actinobacteriota | Chloroflexi | Gemmatimonadota | Verrucomicrobiota | Firmicutes |
| CK | 25.41 | 19.60 | 10.99 | 8.73 | 7.53 | 5.76 | 6.38 | 5.43 | 4.36 | 2.59 |
| NiFe_200_ | 24.99 | 19.37 | 10.39 | 7.62 | 8.69 | 7.31 | 6.51 | 5.04 | 3.91 | 2.83 |
| NiFeS_200_ | 23.27 | 20.34 | 14.15 | 8.84 | 6.94 | 6.85 | 6.07 | 4.49 | 3.87 | 2.51 |
| NiFe_500_ | 24.91 | 19.95 | 11.51 | 7.36 | 6.85 | 6.92 | 7.14 | 5.62 | 3.49 | 3.23 |
| NiFeS_500_ | 21.04 | 21.23 | 10.10 | 9.84 | 5.39 | 8.16 | 8.18 | 5.96 | 3.91 | 2.87 |
| NiFe_800_ | 24.05 | 18.72 | 14.81 | 6.72 | 7.68 | 6.89 | 6.46 | 5.25 | 3.81 | 2.71 |
| NiFeS_800_ | 23.98 | 21.55 | 9.25 | 6.90 | 6.59 | 6.72 | 7.51 | 7.24 | 3.98 | 2.54 |

**Table S6** Relative abundance (%) of the top 6 fungus phyla.

|  | Top 6 fungus phyla | | | | | |
| --- | --- | --- | --- | --- | --- | --- |
|  | Basidiomycota | Ascomycota | Fungi_unclassified | Zygomycota | Chytridiomycota | Glomeromycota |
| CK | 30.91 | 48.49 | 19.00 | 0.91 | 0.41 | 0.23 |
| NiFe_200_ | 52.77 | 40.27 | 5.06 | 1.13 | 0.55 | 0.17 |
| NiFeS_200_ | 43.85 | 47.28 | 7.76 | 0.73 | 0.21 | 0.09 |
| NiFe_500_ | 33.70 | 54.07 | 11.08 | 0.66 | 0.25 | 0.18 |
| NiFeS_500_ | 63.06 | 30.30 | 4.43 | 1.52 | 0.30 | 0.37 |
| NiFe_800_ | 31.19 | 54.64 | 12.24 | 1.08 | 0.61 | 0.21 |
| NiFeS_800_ | 46.21 | 46.07 | 5.65 | 1.34 | 0.53 | 0.17 |

**Table S7** Relative abundance (%) of the top 50 bacterial species.

| Bacterial genus | CK | NiFe_200_ | NiFe_500_ | NiFe_800_ | NiFeS_200_ | NiFeS_500_ | NiFeS_800_ |
| --- | --- | --- | --- | --- | --- | --- | --- |
| Anaeromyxobacter | 8.03 | 7.58 | 8.76 | 12.04 | 11.64 | 7.30 | 6.55 |
| Vicinamibacteraceae_unclassified | 4.45 | 4.50 | 4.54 | 4.37 | 4.71 | 5.45 | 5.08 |
| Vicinamibacterales_unclassified | 3.38 | 3.68 | 4.00 | 3.22 | 3.95 | 3.96 | 3.71 |
| Flavisolibacter | 3.11 | 3.79 | 3.20 | 3.10 | 2.85 | 2.07 | 2.57 |
| RB41 | 2.90 | 2.47 | 1.91 | 2.00 | 2.75 | 2.86 | 3.61 |
| Sphingomonas | 2.33 | 2.33 | 2.26 | 2.66 | 2.55 | 2.27 | 2.68 |
| Gemmatimonadaceae_unclassified | 2.15 | 2.00 | 2.12 | 2.11 | 1.62 | 2.33 | 2.93 |
| Acidobacteriota_unclassified | 2.04 | 2.00 | 2.00 | 2.00 | 2.41 | 2.23 | 1.83 |
| WD2101_soil_group_unclassified | 1.90 | 2.05 | 1.92 | 1.64 | 2.47 | 2.53 | 1.90 |
| SC-I-84_unclassified | 2.33 | 2.65 | 2.39 | 2.06 | 1.39 | 1.38 | 1.53 |
| ADurb.Bin063-1 | 1.86 | 1.74 | 1.35 | 1.74 | 1.87 | 2.06 | 2.03 |
| Gemmataceae_unclassified | 1.52 | 1.34 | 1.25 | 1.22 | 1.61 | 1.57 | 1.03 |
| Gemmatimonas | 1.32 | 1.32 | 1.47 | 1.28 | 1.32 | 1.76 | 2.07 |
| Rhodoplanes | 1.40 | 1.76 | 1.97 | 2.07 | 1.45 | 1.13 | 1.23 |
| Lysobacter | 1.28 | 0.91 | 0.97 | 1.10 | 1.81 | 1.67 | 1.43 |
| KD4-96_unclassified | 1.46 | 1.33 | 1.31 | 1.52 | 1.10 | 1.50 | 1.44 |
| Gemmatimonadota_unclassified | 1.23 | 1.21 | 1.36 | 1.36 | 1.13 | 1.37 | 1.64 |
| Bryobacter | 1.18 | 1.17 | 1.71 | 1.55 | 1.33 | 1.15 | 1.39 |
| Chitinophagaceae_unclassified | 1.24 | 1.48 | 1.05 | 1.22 | 1.72 | 0.70 | 0.99 |
| Roseiflexaceae_unclassified | 0.75 | 1.08 | 0.98 | 0.77 | 1.08 | 1.32 | 1.20 |
| Ramlibacter | 1.00 | 0.96 | 0.87 | 0.82 | 1.52 | 0.64 | 0.88 |
| Candidatus_Solibacter | 0.90 | 1.01 | 1.09 | 1.31 | 0.94 | 0.98 | 1.00 |
| Subgroup_17_unclassified | 1.06 | 0.92 | 0.86 | 0.76 | 0.87 | 0.89 | 0.88 |
| Pedosphaeraceae_unclassified | 0.99 | 1.05 | 0.80 | 0.66 | 0.97 | 0.67 | 0.71 |
| Gemmata | 0.92 | 0.65 | 0.70 | 0.66 | 0.91 | 0.93 | 0.69 |
| Subgroup_7_unclassified | 0.86 | 0.94 | 0.88 | 0.69 | 0.74 | 0.69 | 1.23 |
| Haliangium | 0.80 | 0.84 | 0.93 | 0.80 | 0.68 | 0.77 | 0.77 |
| Pirellulaceae_unclassified | 0.85 | 0.80 | 0.76 | 0.66 | 0.66 | 0.95 | 0.73 |
| Firmicutes_unclassified | 0.58 | 0.57 | 0.74 | 0.74 | 0.46 | 0.81 | 0.60 |
| BIrii41_unclassified | 0.71 | 0.58 | 0.59 | 0.66 | 0.54 | 0.88 | 0.52 |

**Table S8** Relative abundance (%) of the top 50 fungus species.

| Fungus genus | CK | NiFe_200_ | NiFe_500_ | NiFe_800_ | NiFeS_200_ | NiFeS_500_ | NiFeS_800_ |
| --- | --- | --- | --- | --- | --- | --- | --- |
| Tilletia | 23.82 | 25.23 | 25.16 | 20.46 | 18.63 | 17.43 | 20.44 |
| Cryptococcus | 1.42 | 12.29 | 0.75 | 1.50 | 25.72 | 40.74 | 18.06 |
| Fungi_unclassified | 19.00 | 7.76 | 11.08 | 12.24 | 5.06 | 4.43 | 5.65 |
| Ascomycota_unclassified | 8.56 | 5.72 | 8.26 | 5.05 | 4.46 | 2.14 | 4.23 |
| Sordariomycetes_unclassified | 2.62 | 4.42 | 9.20 | 11.59 | 2.48 | 1.49 | 3.29 |
| Hypocreales_unclassified | 7.65 | 5.09 | 7.61 | 3.14 | 2.84 | 1.70 | 1.38 |
| Haematonectria | 3.89 | 4.66 | 2.11 | 2.37 | 2.42 | 2.81 | 2.40 |
| Podospora | 1.83 | 2.78 | 1.18 | 7.25 | 6.74 | 0.21 | 11.16 |
| Trichosporon | 1.57 | 0.87 | 2.87 | 0.30 | 1.02 | 2.05 | 3.63 |
| Paracremonium | 1.30 | 1.04 | 3.52 | 1.33 | 1.38 | 1.01 | 2.80 |
| Trechispora | 1.71 | 1.71 | 1.75 | 3.97 | 2.23 | 1.67 | 1.55 |
| Staphylotrichum | 0.80 | 0.80 | 0.84 | 2.31 | 1.22 | 2.85 | 4.08 |
| Stachybotrys | 0.96 | 1.06 | 1.27 | 1.45 | 1.13 | 1.11 | 1.93 |
| Cladorrhinum | 0.92 | 0.61 | 0.36 | 0.37 | 0.90 | 0.08 | 0.93 |
| Mortierella | 0.91 | 0.73 | 0.66 | 1.08 | 1.10 | 1.52 | 1.34 |
| Microascaceae_unclassified | 1.14 | 1.02 | 1.29 | 1.37 | 1.03 | 0.89 | 1.01 |
| Chaetomium | 1.05 | 1.20 | 1.12 | 1.39 | 1.27 | 0.73 | 0.88 |
| Cyphellophora | 1.48 | 0.52 | 0.74 | 0.74 | 1.50 | 0.58 | 0.57 |
| Fusarium | 0.96 | 0.39 | 0.58 | 1.02 | 0.58 | 1.07 | 0.97 |
| Nectriaceae_unclassified | 1.25 | 0.42 | 1.53 | 1.04 | 0.41 | 4.51 | 0.76 |
| Bionectria | 0.68 | 1.19 | 1.69 | 0.63 | 0.37 | 0.40 | 0.41 |
| Chaetomiaceae_unclassified | 0.79 | 0.80 | 0.73 | 1.12 | 0.76 | 0.61 | 0.67 |
| Khuskia | 0.78 | 0.67 | 0.55 | 0.36 | 0.41 | 0.75 | 0.34 |
| Cochliobolus | 0.50 | 0.84 | 0.61 | 0.32 | 0.89 | 0.45 | 0.40 |
| Pleosporales_unclassified | 0.52 | 0.44 | 0.76 | 0.86 | 0.42 | 0.26 | 0.34 |
| Subulicystidium | 0.11 | 0.87 | 0.21 | 0.29 | 3.36 | 0.10 | 0.11 |
| Agaricales_unclassified | 0.71 | 1.12 | 0.61 | 0.56 | 0.37 | 0.26 | 0.17 |
| Phaeosphaeriaceae_unclassified | 0.60 | 1.14 | 0.32 | 0.65 | 0.75 | 0.25 | 0.36 |
| Conlarium | 0.46 | 0.51 | 0.48 | 0.44 | 0.37 | 0.33 | 0.45 |
| Plectosphaerellaceae_unclassified | 0.38 | 0.45 | 0.42 | 0.43 | 0.49 | 0.31 | 0.26 |

**Table S9** Pearson correlation analysis among soil properties and microbial indexes.

|  |  | pH | EC | TC | TN | TP | SOC | NH^4+^-N | AP | Ni | Fe |
| --- | --- | --- | --- | --- | --- | --- | --- | --- | --- | --- | --- |
| Soil microbial enzyme activity | S-UE | .873** | -.809** | .436** | 0.251 | .419** | -0.109 | -.506** | -0.061 | -.379* | -.305* |
|  | S-SC | -.427** | .544** | -0.113 | -0.063 | 0.010 | 0.121 | 0.212 | -0.025 | -0.201 | 0.107 |
|  | S-CAT | .623** | -.598** | .335* | 0.245 | .399** | -0.268 | -0.223 | 0.181 | -0.285 | -0.163 |
|  | S-NP | -.459** | .534** | -.376* | -.331* | -.427** | .314* | 0.099 | -0.223 | 0.131 | 0.003 |
| Bacteria | chao1 | -0.022 | 0.092 | 0.051 | -0.127 | -0.001 | 0.141 | -0.195 | -0.056 | 0.103 | 0.050 |
|  | shannon | -0.067 | 0.136 | -0.122 | -0.145 | 0.015 | -0.029 | -0.098 | 0.204 | -0.004 | -0.032 |
|  | pielou | -0.144 | .298* | -0.167 | -0.237 | -0.054 | 0.163 | -0.268 | 0.103 | 0.118 | -0.117 |
| Fungus | chao1 | .498** | -.477** | 0.254 | 0.291 | 0.192 | -0.204 | -.406** | -0.261 | -0.094 | -0.274 |
|  | shannon | .564** | -.589** | 0.257 | 0.155 | 0.280 | -0.123 | -.386** | 0.078 | -0.082 | -0.133 |
|  | pielou | .337* | -.327* | 0.125 | -0.084 | 0.176 | -0.030 | -0.270 | 0.086 | 0.095 | -0.045 |
| Bacteria | Proteobacteria | .458** | -.455** | 0.161 | 0.244 | 0.181 | -0.260 | -.384* | 0.187 | -0.256 | -0.116 |
|  | Acidobacteriota | -.435** | .500** | -.299* | -.302* | -0.209 | 0.218 | 0.136 | 0.058 | 0.220 | 0.131 |
|  | Myxococcota | 0.119 | -0.236 | 0.111 | 0.179 | 0.068 | -0.146 | 0.297 | -0.116 | -0.063 | 0.116 |
|  | Planctomycetota | -0.141 | 0.195 | 0.061 | 0.019 | -0.082 | 0.210 | 0.282 | 0.106 | -0.130 | -0.038 |
|  | Bacteroidota | .403** | -.484** | 0.256 | 0.181 | .314* | -.332* | -.344* | 0.169 | -.394** | -0.089 |
|  | Actinobacteriota | -0.160 | 0.197 | -0.099 | -0.197 | -0.209 | 0.218 | 0.102 | -0.194 | 0.286 | -0.220 |
|  | Chloroflexi | -0.279 | .436** | -0.079 | -0.215 | -0.127 | .383* | -0.117 | -0.259 | .300* | -0.228 |
|  | Gemmatimonadota | -0.119 | 0.153 | -0.209 | -0.282 | -0.091 | 0.256 | -.332* | -0.044 | .342* | -0.105 |
|  | Verrucomicrobiota | -0.043 | 0.077 | 0.128 | 0.073 | 0.126 | -0.050 | 0.011 | -0.092 | -0.053 | 0.039 |
| Fungus | Firmicutes | -0.049 | 0.038 | -0.031 | 0.008 | -0.196 | 0.021 | 0.156 | -0.048 | 0.209 | -0.056 |
|  | Basidiomycota | -.681** | .680** | -0.246 | -.377* | -.314* | .434** | .480** | -0.125 | 0.207 | 0.128 |
|  | Ascomycota | .562** | -.558** | 0.290 | 0.108 | .353* | -0.226 | -.545** | 0.047 | -0.089 | -0.027 |
|  | Fungi_unclassified | .577** | -.538** | 0.214 | .447** | .336* | -.318* | -0.200 | 0.162 | -.367* | -0.120 |
|  | Zygomycota | -0.199 | 0.200 | -0.102 | -0.142 | -0.215 | 0.122 | 0.081 | -0.013 | 0.133 | -0.139 |
|  | Chytridiomycota | 0.040 | 0.018 | -0.065 | 0.139 | -.440** | -.315* | 0.040 | -0.006 | 0.018 | -0.269 |
|  | Glomeromycota | -0.016 | -0.030 | -0.134 | 0.090 | -0.140 | -0.232 | -0.058 | 0.120 | 0.093 | 0.026 |
|  | Rozellomycota | .465** | -.478** | 0.147 | 0.222 | 0.260 | -0.269 | -0.198 | 0.032 | -.386** | -0.201 |

**References**

Xu H., Lu L., Yu Z., Lu X., et al. Synergistic interaction between Zn clusters and single-atom Ru in 3D NiFeS-LDH enhance 5-hydroxymethylfurfural electrocatalytic oxidation. Appl. Catal. B: Environ. Energy 2025, 375: 125439

Guan X., Gao X., Avellan A., et al. CuO nanoparticles alter the rhizospheric bacterial community and local nitrogen cycling for wheat grown in a calcareous soil. Environ. Sci. Technol. 2020, 54: 8699-8709.

Logue Jürg B,Stedmon Colin A,Kellerman Anne M et al. Experimental insights into the importance of aquatic bacterial community composition to the degradation of dissolved organic matter. ISME J, 2016, 10: 533-45.

Walters W, Hyde E R, Berglyons D, et al. Improved bacterial 16S rRNA gene (V4 and V45) and fungal internal transcribed spacer marker gene Primers for microbial community surveys. Msystems, 2015, 1(1): e00009-15.

Ken Takai., Koki Horikoshi.Rapid Detection and quantification of members of the archaeal community by quantitative PCR using fluorogenic probes. Applied and environmental microbiology Nov. 2000, p. 5066–5072.
